# Tetra­chlorido(2,3-di-2-pyridylpyrazine-κ^2^ *N* ^1^,*N* ^2^)platinum(IV)

**DOI:** 10.1107/S1600536808007228

**Published:** 2008-03-29

**Authors:** Parvaneh Delir Kheirollahi Nezhad, Fatemeh Azadbakht, Vahid Amani, Hamid Reza Khavasi

**Affiliations:** a Department of Chemistry, Payame Noor University, Iran; b Islamic Azad University, Doroud Branch, Doroud, Iran; c Research Institute in Education, 16 Hojjat Dost Street, Vessal Shirazi Avenue, Tehran, Iran; d Department of Chemistry, Shahid Beheshti University, Tehran 1983963113, Iran

## Abstract

In the title complex, [PtCl_4_(C_14_H_10_N_4_)], the Pt^IV^ atom is six-coordinated in an octa­hedral configuration by two N atoms from one 2,3-di-2-pyridylpyrazine ligand and four terminal Cl atoms. Inter­molecular C—H⋯Cl and C—H⋯N hydrogen bonds stabilize the crystal structure.

## Related literature

For general background, see: Hedin (1886 ▶); Joergensen (1900 ▶); Bajusaz *et al.* (1989 ▶); Vorobevdesyatovskii *et al.* (1991 ▶). For related structures, see: Bokach *et al.* (2003 ▶); Casas *et al.* (2005 ▶); Crowder *et al.* (2004 ▶); Gaballa *et al.* (2003 ▶); Garnovskii *et al.* (2001 ▶); Gonzalez *et al.* (2002 ▶); Hafizovic *et al.* (2006 ▶); Hambley (1986 ▶); Kuduk-Jaworska *et al.* (1988 ▶, 1990 ▶); Junicke *et al.* (1997 ▶); Khripun *et al.* (2006 ▶); Kukushkin *et al.* (1998 ▶); Luzyanin, Haukka *et al.* (2002 ▶); Luzyanin, Kukushkin *et al.* (2002 ▶); Witkowski *et al.* (1997 ▶); Yousefi *et al.* (2007 ▶).
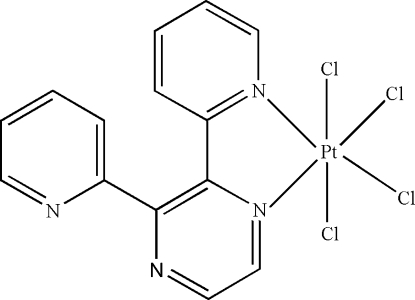


## Experimental

### 

#### Crystal data

[PtCl_4_(C_14_H_10_N_4_)]
*M* *_r_* = 571.14
Orthorhombic, 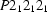

*a* = 6.6849 (4) Å
*b* = 14.9604 (12) Å
*c* = 16.2761 (10) Å
*V* = 1627.75 (19) Å^3^
*Z* = 4
Mo *K*α radiation
μ = 9.28 mm^−1^
*T* = 120 (2) K
0.40 × 0.26 × 0.14 mm

#### Data collection

Stoe IPDSII diffractometer
Absorption correction: numerical (*X-SHAPE* and *X-RED*; Stoe & Cie, 2005 ▶) *T*_min_ = 0.070, *T*_max_ = 0.270
9336 measured reflections
4374 independent reflections
4327 reflections with *I* > 2σ(*I*)
*R*_int_ = 0.064

#### Refinement

*R*[*F* ^2^ > 2σ(*F* ^2^)] = 0.033
*wR*(*F* ^2^) = 0.087
*S* = 1.10
4374 reflections
209 parameters
H-atom parameters constrained
Δρ_max_ = 1.44 e Å^−3^
Δρ_min_ = −1.82 e Å^−3^
Absolute structure: Flack (1983 ▶), 1849 Friedel pairs
Flack parameter: 0.005 (9)

### 

Data collection: *X-AREA* (Stoe & Cie, 2005 ▶); cell refinement: *X-AREA*; data reduction: *X-AREA*; program(s) used to solve structure: *SHELXS97* (Sheldrick, 2008 ▶); program(s) used to refine structure: *SHELXL97* (Sheldrick, 2008 ▶); molecular graphics: *ORTEP-3 for Windows* (Farrugia, 1997 ▶); software used to prepare material for publication: *WinGX* (Farrugia, 1999 ▶).

## Supplementary Material

Crystal structure: contains datablocks I, global. DOI: 10.1107/S1600536808007228/xu2404sup1.cif

Structure factors: contains datablocks I. DOI: 10.1107/S1600536808007228/xu2404Isup2.hkl

Additional supplementary materials:  crystallographic information; 3D view; checkCIF report

## Figures and Tables

**Table:** 

Cl1—Pt1	2.3219 (16)
Cl2—Pt1	2.2945 (16)
Cl3—Pt1	2.3066 (16)
Cl4—Pt1	2.3164 (18)
N1—Pt1	2.036 (5)
N2—Pt1	2.032 (6)

**Table:** 

N2—Pt1—N1	80.4 (2)
N2—Pt1—Cl2	176.45 (16)
N1—Pt1—Cl2	96.12 (17)
N2—Pt1—Cl3	94.15 (16)
N1—Pt1—Cl3	174.20 (18)
Cl2—Pt1—Cl3	89.26 (6)
N2—Pt1—Cl4	90.54 (17)
N1—Pt1—Cl4	89.68 (17)
Cl2—Pt1—Cl4	90.30 (6)
Cl3—Pt1—Cl4	92.45 (7)
N2—Pt1—Cl1	88.17 (17)
N1—Pt1—Cl1	86.68 (17)
Cl2—Pt1—Cl1	90.78 (6)
Cl3—Pt1—Cl1	91.11 (6)
Cl4—Pt1—Cl1	176.30 (7)

**Table 2:** Hydrogen-bond geometry (Å, °)

*D*—H⋯*A*	*D*—H	H⋯*A*	*D*⋯*A*	*D*—H⋯*A*
C1—H1⋯Cl2	0.93	2.68	3.279 (7)	122
C3—H3⋯Cl1^i^	0.93	2.83	3.557 (7)	136
C4—H4⋯N4	0.93	2.59	3.000 (10)	107
C7—H7⋯Cl3	0.93	2.69	3.247 (7)	120
C14—H14⋯Cl1^ii^	0.93	2.74	3.599 (8)	154

Symmetry codes: (i) 
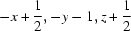
; (ii) 
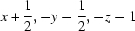
.

**Fig. 1.**
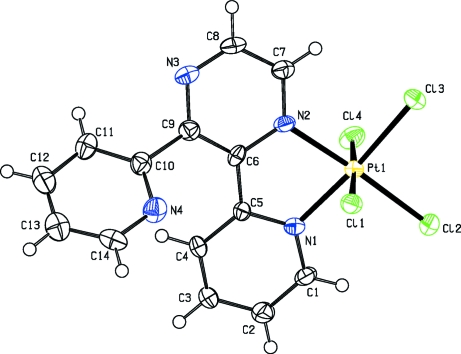
The molecular structure of the title molecule, with the atom-numbering scheme. Displacement ellipsoids are drawn at the 50% probability level.

**Fig. 2.**
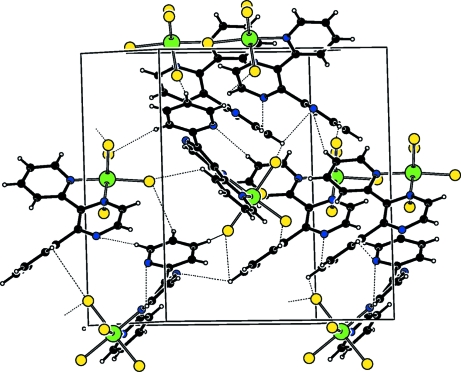
A packing diagram for (I). Hydrogen bonds are shown as dashed lines.

## supplementary crystallographic information

### Comment

Amine platinum(IV) complexes have been known since the end of the last century (Hedin, 1886; Joergensen, 1900). Some of them have cancerostatic properties from which new interest aroused in these complexes (Bajusaz *et al.*, 1989; Vorobevdesyatovskii *et al.*, 1991). Due to the kinetic inertness of hexachloro-platinate(IV), *cis*- and *trans*-[PtC1_4_*L*_2_] complexes (*L*=N, O, P, S donor ligand) were mainly prepared by oxidation reactions of the corresponding platinum(II) complexes [PtCl_2_*L*_2_] (Hedin, 1886; Joergensen, 1900).

Several Pt^IV^ complexes, with formula [PtCl_4_(N—N)], such as [PtCl_4_(bipyi)] (II) (Gaballa *et al.*, 2003), [PtCl_4_(Me_2_bim)] (III) (Casas *et al.*, 2005), [PtCl_4_(bipy)] (IV) (Hambley, 1986), [PtCl_4_(dcbipy)].H_2_O (V) (Hafizovic *et al.*, 2006) and [PtCl_4_(dpk)] (VI) (Crowder *et al.*, 2004) [where bipyi is 2,2'-bipyrimidinyl, Me_2_bim is 1,1'-dimethyl-2,2'-bi-imidazolyl, bipy is 2,2'-bipyridine, dcbipy is 2,2'-bipyridine-5,5'-dicarboxylic acid and dpk is bis(2-pyridyl)ketone] have been synthesized and characterized by single-crystal X-ray diffraction method.

There are also several Pt^IV^ complexes with formula [PtCl_4_*L*_2_], such as *cis*- and *trans*-[PtCl_4_(py)_2_] (VII) (Junicke *et al.*, 1997), *cis*- and *trans*-[PtCl_4_(PzH)_2_] (VIII) (Khripun *et al.*, 2006), *trans*-[PtCl_4_(NH_3_)_2_](1-Mu) (IX) (Witkowski *et al.*, 1997), *trans*-[PtCl_4_(1-Prim)_2_] (*X*) (Kuduk-Jaworska *et al.*, 1988), *cis*-[PtCl_4_(1-Etim)_2_] (XI) (Kuduk-Jaworska *et al.*, 1990), *trans*-[PtCl_4_{NH=C(NMe_2_)OH}_2_] (XII) (Bokach *et al.*, 2003), *trans*-[PtCl_4_{NH=C(Me)ON=CMe_2_}_2_] (XIII) (Kukushkin *et al.*, 1998), *cis*-[PtCl_4_{NH=C(Et)N=CPh_2_}_2_] (XIV) (Garnovskii *et al.*, 2001), *trans*- [PtCl_4_{NH=C(Et)ON=C(OH)Ph}_2_].2DMSO (XV) (Luzyanin, Kukushkin *et al.*, 2002), *trans*-[PtCl_4_{NH=C(OMe)Bu*^t^*}_2_] (XVI) (Gonzalez *et al.*, 2002), *trans*-[PtCl_4_{NH=C(OH)Et}_2_] (XVII) (Luzyanin, Haukka *et al.*, 2002) and *trans*- [PtCl_4_(pz)_2_] (XVIII) (Yousefi *et al.*, 2007) [where PzH is pyrazole, 1-Mu is 1-methyluracil, 1-Prim is 1-propylimidazole 1-Etim is 1-ethylimidazoyl and Pz is pyrazine] have been synthesized and characterized by single-crystal X-ray diffraction method. We report herein the synthesis and crystal structure of the title compound.

In the mononuclear title compound (Fig. 1), the Pt^IV^ atom is six-coordinated in octahedral configuration by two N atoms from one 2,3-di-2-pyridylpyrazine ligand and four terminal Cl atoms. The Pt—Cl and Pt—N bond lengths and angles (Table 1) are in good agreement with the corresponding values in (II), (III) and (V).

In the crystal structure, intermolecular C—H···Cl and C—H···N hydrogen bonds (Table 2) seem to be effective in the stabilization of the crystal structure (Fig. 2).

### Experimental

For the preparation of the title compound, a solution of 2,3-di-2-pyridylpyrazine (0.09 g, 0.37 mmol) in methanol (10 ml) was added to a solution of H_2_PtCl_6_.6H_2_O, (0.20 g, 0.37 mmol) in methanol (10 ml) at room temperature. The suitable crystals for X-ray diffraction experiment were obtained by methanol diffusion in a solution of orange precipitated in DMSO after one week (yield 0.18 g).

### Refinement

H atoms were positioned geometrically with C—H = 0.93 Å and constrained to ride on their parent atoms with *U*_iso_(H)=1.2*U*_eq_(C). The highest peak is 0.4 Å aprat from the Pt1 atom.

### Crystal data

**Table:**

[PtCl_4_(C_14_H_10_N_4_)]	*D*_x_ = 2.331 Mg m^−^^3^
*M**_r_* = 571.14	Melting point: 565-566 K K
Orthorhombic, *P*2_1_2_1_2_1_	Mo *K*α radiation λ = 0.71073 Å
Hall symbol: P 2ac 2ab	Cell parameters from 1050 reflections
*a* = 6.6849 (4) Å	θ = 1.9–29.2º
*b* = 14.9604 (12) Å	µ = 9.28 mm^−^^1^
*c* = 16.2761 (10) Å	*T* = 120 (2) K
*V* = 1627.75 (19) Å^3^	Block, orange
*Z* = 4	0.40 × 0.26 × 0.14 mm
*F*_000_ = 1072	

### Data collection

**Table:**

Stoe IPDSII diffractometer	4374 independent reflections
Radiation source: fine-focus sealed tube	4327 reflections with *I* > 2σ(*I*)
Monochromator: graphite	*R*_int_ = 0.064
Detector resolution: 0.15 mm pixels mm^-1^	θ_max_ = 29.2º
*T* = 120(2) K	θ_min_ = 1.9º
rotation method scans	*h* = −7→9
Absorption correction: Numerical(X-SHAPE and X-RED; Stoe & Cie, 2005)	*k* = −20→17
*T*_min_ = 0.070, *T*_max_ = 0.270	*l* = −22→22
9336 measured reflections	

### Refinement

**Table:**

Refinement on *F*^2^	H-atom parameters constrained
Least-squares matrix: full	*w* = 1/[σ^2^(*F*_o_^2^) + (0.0439*P*)^2^ + 6.2735*P*] where *P* = (*F*_o_^2^ + 2*F*_c_^2^)/3
*R*[*F*^2^ > 2σ(*F*^2^)] = 0.033	(Δ/σ)_max_ = 0.012
*wR*(*F*^2^) = 0.087	Δρ_max_ = 1.44 e Å^−^^3^
*S* = 1.10	Δρ_min_ = −1.82 e Å^−^^3^
4374 reflections	Extinction correction: SHELXL97 (Sheldrick, 2008), Fc^*^=kFc[1+0.001xFc^2^λ^3^/sin(2θ)]^-1/4^
209 parameters	Extinction coefficient: 0.0011 (3)
Primary atom site location: structure-invariant direct methods	Absolute structure: Flack (1983), 1849 Friedel pairs
Secondary atom site location: difference Fourier map	Flack parameter: 0.005 (9)
Hydrogen site location: inferred from neighbouring sites	

### Special details

**Table:**

Geometry. All e.s.d.'s (except the e.s.d. in the dihedral angle between two l.s. planes) are estimated using the full covariance matrix. The cell e.s.d.'s are taken into account individually in the estimation of e.s.d.'s in distances, angles and torsion angles; correlations between e.s.d.'s in cell parameters are only used when they are defined by crystal symmetry. An approximate (isotropic) treatment of cell e.s.d.'s is used for estimating e.s.d.'s involving l.s. planes.
Refinement. Refinement of *F*^2^ against ALL reflections. The weighted *R*-factor *wR* and goodness of fit *S* are based on *F*^2^, conventional *R*-factors *R* are based on *F*, with *F* set to zero for negative *F*^2^. The threshold expression of *F*^2^ > σ(*F*^2^) is used only for calculating *R*-factors(gt) *etc*. and is not relevant to the choice of reflections for refinement. *R*-factors based on *F*^2^ are statistically about twice as large as those based on *F*, and *R*- factors based on ALL data will be even larger.

### Fractional atomic coordinates and isotropic or equivalent isotropic displacement parameters (Å^2^)

**Table:**

	*x*	*y*	*z*	*U*_iso_*/*U*_eq_	
C1	0.2795 (10)	−0.5902 (5)	−0.4681 (4)	0.0286 (13)	
H1	0.3170	−0.6300	−0.5092	0.034*	
C2	0.3870 (11)	−0.5866 (6)	−0.3975 (4)	0.0331 (15)	
H2	0.4966	−0.6241	−0.3901	0.040*	
C3	0.3330 (11)	−0.5272 (6)	−0.3366 (4)	0.0331 (14)	
H3	0.4084	−0.5231	−0.2887	0.040*	
C4	0.1659 (10)	−0.4738 (5)	−0.3472 (4)	0.0275 (12)	
H4	0.1233	−0.4358	−0.3055	0.033*	
C5	0.0622 (8)	−0.4780 (4)	−0.4217 (4)	0.0221 (10)	
C6	−0.1195 (9)	−0.4247 (4)	−0.4420 (4)	0.0218 (11)	
C7	−0.3997 (8)	−0.4210 (5)	−0.5265 (4)	0.0245 (12)	
H7	−0.4677	−0.4395	−0.5733	0.029*	
C8	−0.4867 (9)	−0.3598 (5)	−0.4741 (5)	0.0315 (14)	
H8	−0.6191	−0.3433	−0.4828	0.038*	
C9	−0.1996 (9)	−0.3519 (4)	−0.3978 (4)	0.0244 (12)	
C10	−0.0817 (10)	−0.2972 (4)	−0.3392 (4)	0.0262 (12)	
C11	−0.1610 (11)	−0.2695 (5)	−0.2651 (5)	0.0314 (14)	
H11	−0.2912	−0.2840	−0.2500	0.038*	
C12	−0.0403 (15)	−0.2197 (5)	−0.2143 (5)	0.0403 (17)	
H12	−0.0855	−0.2024	−0.1627	0.048*	
C13	0.1475 (13)	−0.1955 (5)	−0.2404 (5)	0.0372 (16)	
H13	0.2293	−0.1601	−0.2075	0.045*	
C14	0.2123 (12)	−0.2246 (5)	−0.3161 (6)	0.0378 (17)	
H14	0.3391	−0.2073	−0.3334	0.045*	
Cl1	0.1281 (2)	−0.41434 (11)	−0.62538 (10)	0.0272 (3)	
Cl2	0.1441 (2)	−0.63100 (12)	−0.65809 (10)	0.0299 (3)	
Cl3	−0.2674 (2)	−0.52425 (12)	−0.69333 (11)	0.0312 (3)	
Cl4	−0.2288 (3)	−0.65930 (12)	−0.53083 (13)	0.0338 (4)	
N1	0.1199 (7)	−0.5372 (4)	−0.4799 (3)	0.0236 (10)	
N2	−0.2178 (7)	−0.4537 (4)	−0.5100 (4)	0.0240 (10)	
N3	−0.3883 (8)	−0.3242 (4)	−0.4123 (5)	0.0299 (12)	
N4	0.1041 (8)	−0.2763 (4)	−0.3664 (4)	0.0301 (12)	
Pt1	−0.05541 (3)	−0.537445 (15)	−0.582177 (14)	0.02145 (8)	

### Atomic displacement parameters (Å^2^)

**Table:**

	*U*^11^	*U*^22^	*U*^33^	*U*^12^	*U*^13^	*U*^23^
C1	0.027 (3)	0.033 (3)	0.026 (3)	0.013 (3)	0.003 (3)	0.001 (2)
C2	0.026 (3)	0.046 (4)	0.027 (3)	0.005 (3)	0.000 (3)	0.004 (3)
C3	0.028 (3)	0.044 (4)	0.027 (3)	−0.001 (3)	−0.007 (3)	0.005 (3)
C4	0.026 (3)	0.037 (3)	0.020 (3)	0.001 (3)	0.000 (2)	0.002 (2)
C5	0.016 (2)	0.030 (3)	0.020 (2)	−0.005 (2)	0.003 (2)	0.001 (2)
C6	0.018 (2)	0.024 (3)	0.023 (3)	−0.004 (2)	0.001 (2)	0.001 (2)
C7	0.013 (2)	0.029 (3)	0.032 (3)	−0.002 (2)	0.000 (2)	0.004 (2)
C8	0.017 (3)	0.035 (3)	0.043 (4)	0.005 (2)	0.001 (3)	0.004 (3)
C9	0.021 (3)	0.025 (3)	0.027 (3)	0.001 (2)	0.002 (2)	−0.002 (2)
C10	0.023 (3)	0.027 (3)	0.028 (3)	0.004 (2)	0.003 (2)	0.003 (2)
C11	0.032 (3)	0.029 (3)	0.033 (3)	0.002 (3)	0.006 (3)	−0.001 (3)
C12	0.057 (5)	0.033 (3)	0.031 (3)	0.008 (4)	−0.005 (4)	−0.011 (3)
C13	0.039 (4)	0.034 (4)	0.038 (4)	0.001 (3)	−0.005 (3)	−0.004 (3)
C14	0.034 (4)	0.031 (4)	0.049 (5)	−0.005 (3)	−0.004 (3)	0.004 (3)
Cl1	0.0207 (6)	0.0351 (8)	0.0258 (7)	−0.0030 (6)	−0.0009 (6)	0.0029 (6)
Cl2	0.0255 (7)	0.0362 (8)	0.0278 (7)	0.0049 (6)	0.0011 (6)	−0.0056 (6)
Cl3	0.0239 (6)	0.0386 (9)	0.0312 (7)	0.0024 (6)	−0.0081 (6)	−0.0064 (7)
Cl4	0.0282 (7)	0.0296 (8)	0.0437 (9)	−0.0046 (6)	0.0073 (7)	−0.0032 (7)
N1	0.0149 (19)	0.033 (3)	0.022 (2)	−0.002 (2)	−0.0005 (17)	0.003 (2)
N2	0.0113 (19)	0.029 (3)	0.031 (3)	0.0035 (19)	0.0002 (18)	0.002 (2)
N3	0.019 (2)	0.030 (3)	0.040 (3)	0.0024 (19)	0.001 (2)	−0.002 (3)
N4	0.025 (3)	0.034 (3)	0.031 (3)	−0.007 (2)	0.002 (2)	0.001 (2)
Pt1	0.01531 (11)	0.02622 (12)	0.02281 (12)	0.00055 (8)	−0.00071 (8)	−0.00174 (9)

### Geometric parameters (Å, °)

**Table:**

C1—N1	1.342 (8)	C9—N3	1.349 (8)
C1—C2	1.357 (10)	C9—C10	1.484 (9)
C1—H1	0.9300	C10—N4	1.355 (9)
C2—C3	1.379 (11)	C10—C11	1.382 (10)
C2—H2	0.9300	C11—C12	1.373 (11)
C3—C4	1.385 (9)	C11—H11	0.9300
C3—H3	0.9300	C12—C13	1.374 (13)
C4—C5	1.398 (8)	C12—H12	0.9300
C4—H4	0.9300	C13—C14	1.377 (12)
C5—N1	1.353 (8)	C13—H13	0.9300
C5—C6	1.490 (8)	C14—N4	1.339 (10)
C6—N2	1.359 (8)	C14—H14	0.9300
C6—C9	1.411 (9)	Cl1—Pt1	2.3219 (16)
C7—N2	1.338 (7)	Cl2—Pt1	2.2945 (16)
C7—C8	1.379 (10)	Cl3—Pt1	2.3066 (16)
C7—H7	0.9300	Cl4—Pt1	2.3164 (18)
C8—N3	1.315 (10)	N1—Pt1	2.036 (5)
C8—H8	0.9300	N2—Pt1	2.032 (6)
			
N1—C1—C2	121.2 (7)	C11—C12—C13	119.6 (8)
N1—C1—H1	119.4	C11—C12—H12	120.2
C2—C1—H1	119.4	C13—C12—H12	120.2
C1—C2—C3	119.7 (7)	C12—C13—C14	118.7 (8)
C1—C2—H2	120.2	C12—C13—H13	120.6
C3—C2—H2	120.2	C14—C13—H13	120.6
C2—C3—C4	119.6 (7)	N4—C14—C13	124.1 (8)
C2—C3—H3	120.2	N4—C14—H14	118.0
C4—C3—H3	120.2	C13—C14—H14	118.0
C3—C4—C5	118.8 (6)	C1—N1—C5	120.9 (6)
C3—C4—H4	120.6	C1—N1—Pt1	125.0 (5)
C5—C4—H4	120.6	C5—N1—Pt1	114.1 (4)
N1—C5—C4	119.7 (6)	C7—N2—C6	119.1 (6)
N1—C5—C6	115.3 (5)	C7—N2—Pt1	126.5 (5)
C4—C5—C6	124.9 (6)	C6—N2—Pt1	114.2 (4)
N2—C6—C9	118.6 (6)	C8—N3—C9	118.5 (6)
N2—C6—C5	113.8 (5)	C14—N4—C10	115.4 (7)
C9—C6—C5	127.6 (6)	N2—Pt1—N1	80.4 (2)
N2—C7—C8	120.1 (7)	N2—Pt1—Cl2	176.45 (16)
N2—C7—H7	119.9	N1—Pt1—Cl2	96.12 (17)
C8—C7—H7	119.9	N2—Pt1—Cl3	94.15 (16)
N3—C8—C7	122.1 (6)	N1—Pt1—Cl3	174.20 (18)
N3—C8—H8	119.0	Cl2—Pt1—Cl3	89.26 (6)
C7—C8—H8	119.0	N2—Pt1—Cl4	90.54 (17)
N3—C9—C6	120.2 (6)	N1—Pt1—Cl4	89.68 (17)
N3—C9—C10	116.1 (6)	Cl2—Pt1—Cl4	90.30 (6)
C6—C9—C10	123.5 (6)	Cl3—Pt1—Cl4	92.45 (7)
N4—C10—C11	124.6 (7)	N2—Pt1—Cl1	88.17 (17)
N4—C10—C9	113.8 (6)	N1—Pt1—Cl1	86.68 (17)
C11—C10—C9	121.5 (6)	Cl2—Pt1—Cl1	90.78 (6)
C12—C11—C10	117.6 (7)	Cl3—Pt1—Cl1	91.11 (6)
C12—C11—H11	121.2	Cl4—Pt1—Cl1	176.30 (7)
C10—C11—H11	121.2		
			
N1—C1—C2—C3	−0.5 (12)	C8—C7—N2—C6	−1.0 (10)
C1—C2—C3—C4	2.0 (11)	C8—C7—N2—Pt1	−175.7 (5)
C2—C3—C4—C5	−3.6 (10)	C9—C6—N2—C7	−9.1 (9)
C3—C4—C5—N1	3.6 (9)	C5—C6—N2—C7	169.0 (5)
C3—C4—C5—C6	180.0 (6)	C9—C6—N2—Pt1	166.1 (5)
N1—C5—C6—N2	9.9 (8)	C5—C6—N2—Pt1	−15.7 (7)
C4—C5—C6—N2	−166.6 (6)	C7—C8—N3—C9	−3.2 (11)
N1—C5—C6—C9	−172.2 (6)	C6—C9—N3—C8	−7.3 (10)
C4—C5—C6—C9	11.3 (10)	C10—C9—N3—C8	167.8 (7)
N2—C7—C8—N3	7.7 (11)	C13—C14—N4—C10	2.0 (11)
N2—C6—C9—N3	13.6 (10)	C11—C10—N4—C14	−0.5 (10)
C5—C6—C9—N3	−164.2 (6)	C9—C10—N4—C14	178.0 (6)
N2—C6—C9—C10	−161.1 (6)	C7—N2—Pt1—N1	−172.4 (6)
C5—C6—C9—C10	21.1 (10)	C6—N2—Pt1—N1	12.8 (5)
N3—C9—C10—N4	−132.2 (7)	C7—N2—Pt1—Cl3	9.7 (6)
C6—C9—C10—N4	42.7 (9)	C6—N2—Pt1—Cl3	−165.2 (4)
N3—C9—C10—C11	46.4 (9)	C7—N2—Pt1—Cl4	−82.8 (5)
C6—C9—C10—C11	−138.7 (7)	C6—N2—Pt1—Cl4	102.3 (4)
N4—C10—C11—C12	−2.2 (11)	C7—N2—Pt1—Cl1	100.7 (5)
C9—C10—C11—C12	179.4 (7)	C6—N2—Pt1—Cl1	−74.2 (4)
C10—C11—C12—C13	3.4 (11)	C1—N1—Pt1—N2	172.5 (6)
C11—C12—C13—C14	−2.1 (12)	C5—N1—Pt1—N2	−7.1 (4)
C12—C13—C14—N4	−0.7 (12)	C1—N1—Pt1—Cl2	−8.3 (6)
C2—C1—N1—C5	0.5 (11)	C5—N1—Pt1—Cl2	172.0 (4)
C2—C1—N1—Pt1	−179.1 (6)	C1—N1—Pt1—Cl4	81.9 (6)
C4—C5—N1—C1	−2.1 (9)	C5—N1—Pt1—Cl4	−97.7 (4)
C6—C5—N1—C1	−178.8 (6)	C1—N1—Pt1—Cl1	−98.8 (6)
C4—C5—N1—Pt1	177.5 (5)	C5—N1—Pt1—Cl1	81.6 (4)
C6—C5—N1—Pt1	0.8 (6)		

### Hydrogen-bond geometry (Å, °)

**Table:**

*D*—H···*A*	*D*—H	H···*A*	*D*···*A*	*D*—H···*A*
C1—H1···Cl2	0.93	2.68	3.279 (7)	122
C3—H3···Cl1^i^	0.93	2.83	3.557 (7)	136
C4—H4···N4	0.93	2.59	3.000 (10)	107
C7—H7···Cl3	0.93	2.69	3.247 (7)	120
C14—H14···Cl1^ii^	0.93	2.74	3.599 (8)	154

Symmetry codes: (i) −*x*+1/2, −*y*−1, *z*+1/2; (ii) *x*+1/2, −*y*−1/2, −*z*−1.
